# Using physiologically-based pharmacokinetic modeling to assess the efficacy of glove materials in reducing internal doses and potential hazards of N-methylpyrrolidone during paint stripping

**DOI:** 10.1038/s41370-020-0218-2

**Published:** 2020-03-09

**Authors:** C. R. Kirman

**Affiliations:** Summit Toxicology, LLP, PO Box 3209, Bozeman, MT 59715 USA

**Keywords:** PBPK, Gloves, Efficacy, Risk assessment

## Abstract

A refined risk assessment was conducted to evaluate the efficacy of different glove materials in reducing the potential hazards associated with using paint strippers containing N-methylpyrrolidone (NMP) under the scenarios defined by USEPA’s TSCA risk assessment. Three categories of gloves were identified based on measured permeation rates for NMP: (1) minimal protection; (2) moderate protection; and (3) maximal protection. Simulations for eight acute and chronic occupational exposure scenarios identified by USEPA as having a potential hazard (i.e., margins of exposure, MOE, <30) were reassessed for each glove category using PBPK modeling to predict peak (Cmax) and cumulative (AUC) internal doses of NMP. For the acute assessment, the refined MOE values were ≥30 for half of the scenarios for gloves from the moderate protection group category, and all of the scenarios for gloves from the maximal protection category. For the chronic assessment, the refined MOE values were ≥30 for all scenarios except one for gloves from the maximal protection category. The results of this assessment indicate that: (1) the degree of protection provided by gloves from NMP permeation can vary widely depending upon the glove material, NMP formulation, and internal dose measure (with calculated glove protection factors ranging from 1.1 to 1900); and (2) NMP-containing paint strippers can be used safely when appropriate PPE are used. As such, these results can be used to support risk-reduction methods (e.g., product labeling, MSDS instructions on use of appropriate glove materials) as alternatives to banning NMP use under TSCA.

## Introduction

The Toxic Substances Control Act (TSCA), originally passed in 1976 and amended in 2016, provides EPA with authority to require reporting, record-keeping and testing requirements, and restrictions relating to chemical substances and/or mixtures. In March of 2015, USEPA released its final risk assessment for N-methylpyrrolidone (NMP) used in paint strippers under TSCA [1]. In this assessment, USEPA evaluated acute and chronic exposure scenarios to workers and consumers using NMP-containing paint strippers. To support their assessment, USEPA relied upon several state-of-the-science tools/models, including physiologically based pharmacokinetic (PBPK) modeling, benchmark dose modeling, as well as a consideration of personal protective equipment (PPE) to reduce potential exposures. With respect to glove use, USEPA concluded the following:

“*The use of gloves was determined to be effective in reducing modeled estimates of exposure, as demonstrated by the higher MOEs. For chronic exposure, gloves may not provide sufficient protection in all scenarios. More importantly, not all glove types are effective in protecting against NMP exposure. USEPA did not evaluate glove efficacy, however California DOH recommends the use of gloves made of butyl rubber or laminated polyethylene/EVOH2*.”

The efficacy of glove materials is an important factor to consider when evaluating methods for mitigating potential hazards from NMP exposure. Glove materials vary greatly in their effectiveness as a barrier to NMP, with measured permeation rates spanning nearly three orders of magnitude [2–4]. For most of the exposure scenarios assessed by USEPA (all consumer scenarios, all nearby worker scenarios, and most central tendency worker scenarios) the margins of exposures (MOEs) calculated were deemed acceptable (i.e., MOE ≥ 30). For eight central tendency and high-end worker scenarios, a potential unacceptable hazard was identified (i.e., MOE < 30) [1]. The goal of this work is to conduct a refined risk assessment for NMP use in paint strippers for these eight scenarios. Specifically, the efficacy of different glove materials was assessed using PBPK modeling to quantify the degree of protection offered under the conditions defined by the exposure scenarios developed by USEPA in their TSCA risk assessment for NMP.

## Methods

USEPA’s risk assessment for NMP utilized a margin of exposure (MOE) approach to characterize potential hazards. Use of PBPK modeling by USEPA permits this approach to be assessed in terms of internal dose estimates for toxicity and exposure:

1 $${\mathrm{MOE}} = {\mathrm{ID}}_{\mathrm{TA}}/{\mathrm{ID}}_{{\mathrm{EA}}},$$

Where,

- MOE = Margin of exposure (unitless);
- ID_TA_ = Internal dose for the point of departure (POD) from the toxicity assessment for NMP (mg/L or mg h/L); and
- ID_EA_ = Internal dose from the exposure assessment for paint stripping scenarios for NMP (mg/L or mg h/L).

Internal doses of NMP used by USEPA in their assessment include peak blood concentrations (Cmax, mg/L) to assess acute exposures, and area under the curve (AUC, mg h/L) for NMP in blood to assess chronic exposures. MOE values for all consumer, nearby occupational, and low-level occupational scenarios were calculated to be 30 or higher, where 30 is identified as an acceptable MOE value by USEPA [i.e., no concern for adverse effects of NMP if exposure (ID_EA_) is at least 30-fold lower than toxicity (ID_TA_)]. These scenarios are not reassessed here. However, MOE values calculated for eight mid- and high-exposure level occupational scenarios were <30, with some calculated to be as low as 0.1. A summary of the results for the occupational scenarios (without gloves) from USEPA’s risk assessment with MOE values <30 is provided in Table 1.

**Table 1:** Select no-glove occupational exposure scenarios for NMP paint stripper use under TSCA [1]^a^.

Exposure scenario	Exposure level (NMP liquid exposure)	Respirator use	Estimated margin of exposure	
			Acute	Chronic
Miscellaneous stripping	Mid-range (NMP Solution)	−	12.7	5.4
		+	13.7	5.9
	High-end (Neat NMP)	−	0.7	0.1
		+	0.7	0.1
Graffiti removal	Mid-range (NMP Solution)	−	14.1	6.1
		+	14.1	6.1
	High-end (Neat NMP)	−	0.7	0.1
		+	0.7	0.1

^a^Only exposure scenarios identified with potential hazard (i.e., MOE < 30) are included here.

USEPA’s toxicity and exposure assessment for NMP, along with a description of the refinements made for the dermal liquid pathway, are summarized below.

### Summary of USEPA’s toxicity assessment for NMP

The toxicity of NMP in laboratory animals has been well studied, with developmental effects consistently identified as the most sensitive endpoint for risk assessment purposes [1, 5–7]. The parent compound, rather than one of its metabolites, has been identified as the likely developmental toxin based on the results of in vivo and in vitro studies in rats [8, 9]. This conclusion supports the use of the parent chemical in blood as an appropriate measure of internal dose for characterizing the dose–response relationships for developmental effects.

USEPA’s toxicity assessment was adopted unchanged for this assessment, so that the focus remains on the impact glove materials on potential hazards. USEPA modified a PBPK model developed for NMP in rats [6] for the purposes of: (1) characterizing the dose–response relationship for developmental effects in terms of internal dose; and (2) permitting the use of dose–response data collected for oral and inhalation NMP exposures in a combined manner. Minor corrections and modifications were made to the model code, as described in Appendix I of USEPA’s assessment [1]. USEPA assessed endpoints for both acute and chronic exposures to NMP, as summarized below and in Table 2.

- *Acute exposures*—For acute exposures, USEPA identified fetal resorptions observed in rats following oral gavage exposures to NMP [10], but not after inhalation exposures to NMP [11] as the key endpoint of interest. The dose–response data for both oral and inhalation exposures were combined and assessed in terms of peak concentration of NMP in maternal blood (Cmax, mg/L). Based on the best fitting dose–response model (Hill) and a 1% benchmark response rate, a point of departure value (BMDL01) of 216 mg/L was determined for fetal resorptions.
- *Chronic exposures*—For chronic exposures, USEPA identified decreased fetal body weight observed in rats following inhalation exposures to NMP [11] as the key endpoint of interest. The dose–response data for inhalation exposures were assessed in terms of cumulative internal dose of NMP in maternal rat blood (AUC, mg h/L). Based on the best fitting dose–response model (linear) and a 5% benchmark response rate, a point of departure value (BMDL05) of 411 mg h/L was determined for fetal body weight decrements.

**Table 2:** Summary of NMP toxicity values expressed in terms of internal dose.

Assessment decision	Acute assessment	Chronic assessment
Endpoint (key study)	Increased incidence of fetal resorptions in rats (Saillenfait et al. [10, 11])	Decreased fetal body weights in rats (Saillenfait et al. [11])
Internal dose	Cmax	AUC
Benchmark dose model	Hill	Linear
Benchmark response rate	1%	5%
Point of departure	BMDL01 = 216 mg/L	BMDL05 = 411 mg h/L

The two point of departure values summarized here (216 mg/L and 411 mg h/L) serve as the numerators (ID_TA_) for calculating acute and chronic MOE values in Eq. (1). Additional analyses (i.e., use of other endpoints, dose measures, dose–response models) were performed by USEPA in support of the points of departure selected. Uncertainties associated with the selected points of departure are summarized in the discussion section.

### Summary of USEPA exposure assessment for NMP

USEPA’s exposure assessment included consideration of three exposure pathways: (1) inhalation exposures to NMP vapors; (2) dermal exposure to NMP vapors; and (3) dermal exposure to NMP liquid. For this assessment, the first two exposure pathways remain unchanged, while the later pathway was refined to permit a characterization of the effect of different glove materials on estimated internal doses of NMP. Acute exposures were assessed for both occupational and consumer scenarios, while chronic exposures were assessed only for occupational scenarios, since consumer scenarios are expected to be associated with short-term specific tasks. Occupational scenarios include miscellaneous stripping (low, mid, high exposures) and graffiti removal (low, mid, high exposures). Consumer scenarios include brush on (indirect, mid, and high exposures) and spray on (indirect and high exposures) applications either in a workshop or bathroom. The use of PPE (respirator and/or gloves) was varied to determine how this might affect exposure in both occupational and consumer scenarios. As stated above, only a subset of these scenarios (i.e., MOE < 30) are considered here (as listed in Table 1).

For both acute and chronic exposure scenarios, USEPA relied upon a human PBPK model for NMP to calculate internal doses (i.e., corresponding to the denominator, ID_EA_, in Eq. (1)). Internal dose estimates are expected to better represent exposures related to potential adverse effects [12]. The human PBPK model for NMP allowed for aggregating exposures across multiple exposure routes/pathways, specifically dermal, vapor-through-skin, and inhalation exposures. The PBPK model was based on a published, peer-reviewed model [6] that was modified and validated for use by USEPA to support their risk assessment.

### Exposure assessment refinements for glove use

A literature search was conducted to identify key studies and datasets for evaluating the permeation of NMP through glove materials. Three studies were identified and are summarized briefly below.

Zellers and Sulewski [2] assessed the temperature dependence of NMP permeation through different glove materials used in microelectronics fabrication facilities (ASTM F739-85 permeation test method). The butyl-rubber glove showed no breakthrough after 4 h of exposure at any temperature, and NMP permeation was not detected at any time point. Breakthrough times and steady-state permeation rates for the other gloves, and their temperature dependence, were described. Permeation rates for NMP using glove materials other than butyl rubber ranged from 6 to 19 µg/cm^2^/min.

Stull et al. [3] conducted a multiphase study to evaluate how gloves resist multichemical-based paint stripping formulations, including those that contain NMP. Twenty different glove types were identified for initial evaluation. Degradation resistance screening was carried out for each glove style and paint stripping formulation, and gloves least affected were identified. Gloves were then evaluated for their resistance to permeation using continuous contact testing (ASTM Test Method F 739), with those showing extensive permeation undergoing further testing for intermittent contact (modified form of ASTM Test Method F 1383). These results were used to select glove styles to be tested using commercially available paint stripping products. Gloves made of plastic laminate and butyl rubber were the most effective against the majority of paint strippers. The authors concluded that more glove styles resisted permeation by NMP and dibasic ester-based paint strippers than alternative solvent-based paint stripers such as methylene chloride, methanol, isopropanol, acetone, and toluene. The authors also found that decreased contact time caused relatively little change in permeation resistance and that the surrogate paint stripper data did not always accurately predict resistance to the commercial paint stripper formulations. Permeation rates for NMP using different glove materials were reported to vary by nearly three orders of magnitude (<0.1–94 µg/cm^2^/min).

Crook and Simpson [4] tested 20 glove types for their permeability to neat NMP and NMP-containing formulations. Initial screening of gloves was performed by visual inspection and gravimetric evaluation of solvent uptake over a 4-h period. In the second phase, gloves were evaluated for resistance to NMP permeation. Butyl rubber and laminate gloves generally offered the greatest degree of protection from NMP permeation. Moderate permeation rates were observed for polyethylene gloves. High permeation rates were observed for latex and nitrile gloves, with some gloves exhibiting acute failure. Some variation in results across brands for the same glove type and NMP formulations was observed. Overall, permeation rates for NMP using different glove materials in this study were reported to vary by more than two orders of magnitude (<0.1 to >34 µg/cm^2^/min).

NMP steady-state permeation rates as reported in the permeation studies (i.e., NMP flux, µg/cm^2^ min) from these three studies are summarized in Table 3, and were used to calculate permeability coefficient (Kp, cm/h) values, which are used to characterize dermal uptake in the PBPK model, using the following equation:

2 $${\mathrm{Kp}} = \frac{{{\mathrm{PR}}}}{C} \times {\mathrm{CF}}$$

Where, Kp = permeability coefficient (cm/h); PR = permeation rate (µg/cm^2^/min; Table 3) *C* = NMP test concentration (mg/L; Table 3) CF = conversion factor (0.001 mg/µg × 1000 cm^3^/L × 60 min/h).

**Table 3:** Summary of NMP permeation rates for various glove materials.

Glove category	Glove material	Glove brand	Test Material	Permeation rate (µg/ cm^b^/min)^a^	NMP test concentration (mg/cm^3^)	Permeability coefficient (cm/h)^b^	Reference
Minimal protection	Refinishing gloves (natural rubber)	Thompson & Forby	NMP Formulation IV	**94**	773	0.0073	Stull et al. [3]
			Stripper IV-B	**14**	381	0.0022	
			NMP Formulation V	7.7	515	0.00090	
			Stripper IV-A	6.6	690	0.00057	
			NMP Formulation VI	0.19	371	0.000031	
	Nitrile	Kimberly-Clark Safeskin 52002M	NMP	>34	1030	0.0020	Crook and Simpson [4]
		Ansell Solvex 37–675	NMP	32	1030	0.0019	
	Latex	Ansell Conform 69–150	NMP	>26	1030	0.0015	
		Ansell	NMP	39	1030	0.0023	Zellers and Sulewski [2]
		Edmont Puretek	NMP	16	1030	0.00093	
	Latex/neoprene/ nitrile	Pioneer Trionic	NMP	17	1030	0.00099	
Moderate protection	Polyethylene	Ansell Profood 35–405	Graffiti Gone CR-GR1	**1.6**	464	0.00021	Crook and Simpson [4]
			NMP	1.2	1030	0.000070	
Maximum protection	Butyl	North	Stripper IV-B	**0.3**	381	0.000047	Stull et al. [3]
		KCL Butoject 898	NMP	<0.1	1030	0.0000029	Crook and Simpson [4]
			Graffiti Gone CR-GR1	<0.1	464	0.0000065	
		Comasec	NMP Formulation IV	<0.1	773	0.0000039	Stull et al. [3]
			NMP Formulation V	<0.1	515	0.0000058	
			NMP Formulation VI	<0.1	371	0.0000081	
		Guardian	NMP Formulation IV	<0.1	773	0.0000039	
			NMP Formulation V	<0.1	515	0.0000058	
			NMP Formulation VI	<0.1	371	0.0000081	
		North	NMP Formulation IV	<0.1	773	0.0000039	
			NMP Formulation V	<0.1	515	0.0000058	
			NMP Formulation VI	<0.1	371	0.0000081	
			Stripper IV-A	<0.1	690	0.0000043	
			NMP	Not detected	–	–	Zellers and Sulewski [2]
	Laminate	North Silver Shield	NMP	<0.1	1030	0.0000029	Crook and Simpson [4]
		North Silver Shield	Graffiti Gone CR-GR1	<0.1	464	0.0000065	
		Safety 4	NMP Formulation IV	<0.1	773	0.0000039	Stull et al. [3]
			NMP Formulation V	<0.1	515	0.0000058	
			NMP Formulation VI	<0.1	371	0.0000081	
			Stripper IV-A	<0.1	690	0.0000043	
			Stripper IV-B	<0.1	381	0.0000078	

^a^Maximum values for each category in bold were used to represent the glove group for PBPK simulations.

^b^Permeability coefficient = Permeation rate/NMP concentration × conversion factor (1 mg/1000 µg) × (1000 cm^3^/L); For nondetect permeation rates (e.g., <0.1), a value of ½ the detection limit was used (e.g., 0.05).

Based on the data available for NMP permeation, three categories of glove materials were identified: (1) minimal protection (materials with permeation rates greater than 2 µg/cm^2^ min); (2) moderate protection (materials with permeation rates between 1 and 2 µg/cm^2^/min); and (3) maximal protection (materials permeation rates ≤0.3 µg/cm^2^/min) (Table 3).

Net permeability coefficients for gloved hands were modeled as a multi-layered barrier consistent with Fick’s law using the following equation, adapted from Solovyov and Goldman [13]:

3 $${\mathrm{Kp}}_{{\mathrm{net}}} = \frac{1}{{\frac{1}{{{\mathrm{Kp}}_{{\mathrm{skin}}}}} + \frac{1}{{{\mathrm{Kp}}_{{\mathrm{glove}}}}}}}$$

Where, Kp_net_ = net permeability coefficient for NMP through gloved skin (cm/h; Table 4); Kp_skin_ = permeability coefficient for NMP through skin (0.00205 cm/h for neat NMP; 0.000478 cm/h for NMP solutions; USEPA, 2015); and Kp_glove_ = permeability coefficient for NMP through glove material (cm/h; Table 3).

**Table 4:** NMP glove protection factors calculated for different glove materials.

Liquid NMP exposure	Glove category	Net permeability coefficient for gloved skin (cm/h)^a^	Protection factors for specific internal dose measures^b^	
			Cmax	AUC
NMP Solution	Minimal Protection	0.00038 (0.000029–0.00045)	1.3 (1.1–18)	1.3 (1.1–18)
	Moderate Protection	0.00011 (0.000061–0.00015)	4.7 (3.5–8.4)	4.9 (3.6–8.7)
	Maximal Protection	0.0000076 (0.0000029–0.000043)	68 (12–180)	71 (12–190)
Neat NMP	Minimal Protection	0.00098 (0.000030–0.0016)	2.5 (1.3–130)	3.0 (1.4–180)
	Moderate Protection	0.00013 (0.000068–0.00019)	28 (18–56)	39 (26–78)
	Maximal Protection	0.0000077 (0.0000029–0.000046)	510 (83–1400)	720 (120–1900)

^a^Value reflects the mean calculated for the glove category using Eq. (3). Range of values indicated in parentheses reflects the minimum and maximum Kp values for the glove category.

^b^Value reflects the mean calculated for the glove category using Eq. (4). Range of values indicated in parentheses reflects the minimum and maximum values (based on Kp range) for the glove category, and the minimum and maximum internal doses across exposure scenarios.

Use of this equation conservatively assumes that there is no significant accumulation of NMP liquid between glove and skin, which would serve to reduce the concentration gradient and net permeation of NMP across the glove material. Furthermore, in applying the Kp_net_ term to simulations, the skin surface area exposed to NMP, which was defined as the entire glove surface area on one or both hands (depending on the scenario), was assumed to remain constant and unchanged (i.e., because glove use is modeled herein to affect the rate of absorption, no change was made to skin surface area, as modeled by USEPA in their assessment). The mean and range of net permeability coefficients identified for each glove category (Table 4) were used to characterize NMP glove permeation in this risk assessment.

No changes were made to the PBPK model structure, parameter values (other than the refined Kp values), or assumptions defined by USEPA [1]. For the eight exposures scenarios resulting in MOE values < 30 (Table 1), the PBPK model was used to simulate the impact of the Kp_net_ values for gloved skin using different gloves types to assess their effect on predicted internal dose estimates, both with and without the use of a respirator. The internal doses and MOE values were compared with the values calculated by USEPA for exposure scenarios without gloves to assess glove material efficacy. To isolate the impact of gloves on the dermal liquid exposure pathway, PBPK simulations were also run for the eight occupational scenarios for the dermal liquid pathway alone (i.e., excluding inhalation and dermal vapor pathways) to calculate glove protection factor (PF) values for each glove category using the equation below:

4 $${\mathrm{PF}} = {\mathrm{ID}}_{{\mathrm{no}}\,{\mathrm{gloves}}}/{\mathrm{ID}}_{{\mathrm{gloves}}}$$

Where, PF = Protection factor (unitless); ID_no gloves_ = Internal dose for occupational simulations of the dermal liquid pathway without gloves (Cmax for NMP in blood, mg/L; AUC for NMP in blood, mg h/L); and ID_gloves_ = Internal dose for occupational simulations of the dermal liquid pathway with gloves (Cmax for NMP in blood, mg/L; AUC for NMP in blood, mg h/L).

## Results

**Fig. 1:**
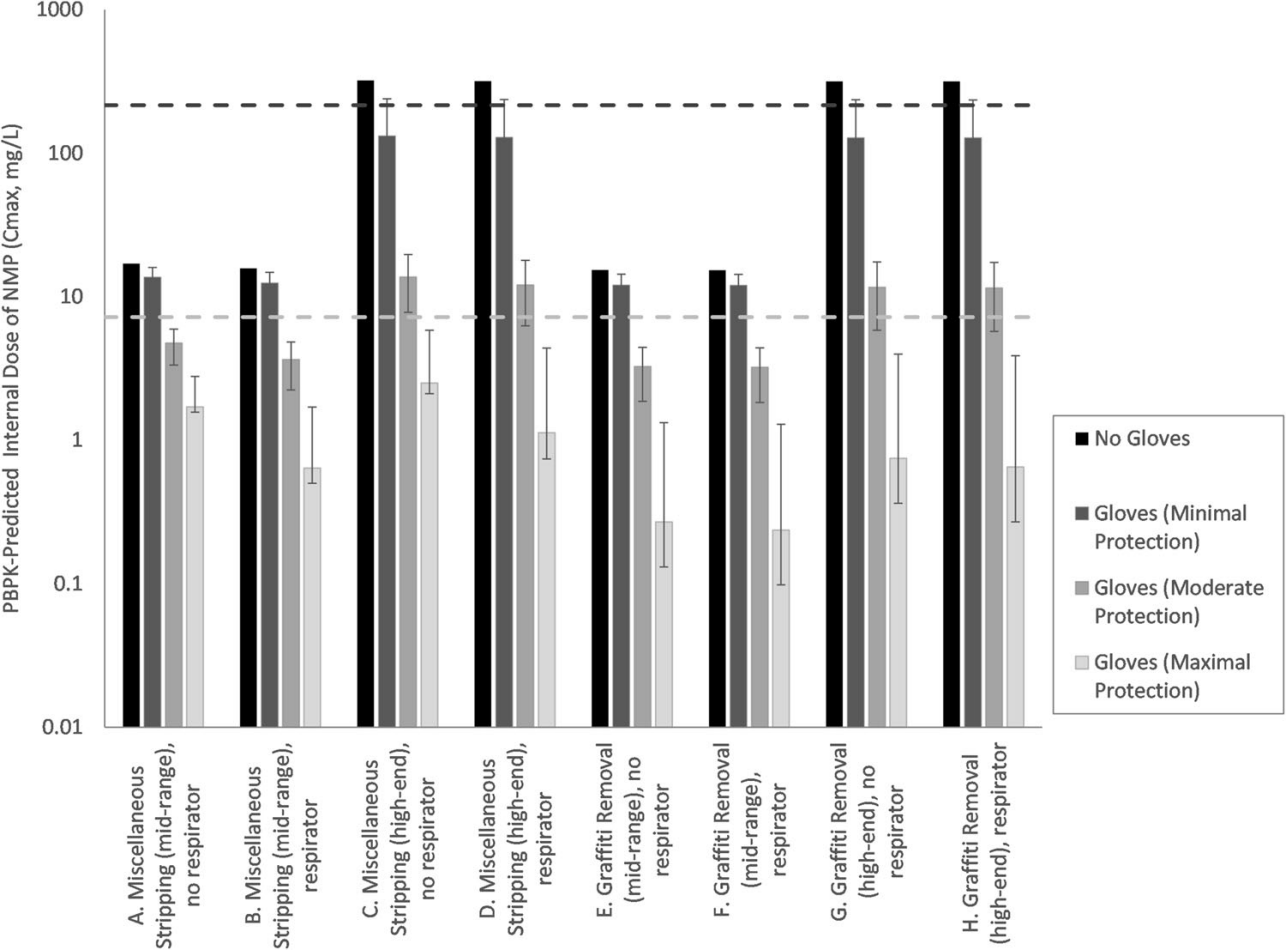
Internal dose estimates for acute exposure scenarios using different glove types. Columns indicate the mean value for the glove category, error bars indicate the range for the glove category (based on range of Kp_net_ values).

MOE results for the acute exposure scenarios are provided in Fig. 1. MOE values calculated for the moderate and maximum protection glove categories exhibit substantial improvement over the no-glove scenario values calculated by USEPA, while those calculated for minimum protection glove categories were minimally changed. Specifically, MOE values (rounded to two significant figures) calculated by USEPA for no-glove scenarios ranged from 0.7 to 14, while the mean MOE values calculated for use of minimal, moderate, and maximum protection glove types range across scenarios from 1.6 to 18, 16 to 67, and 86 to 910, respectively.

**Fig. 2:**
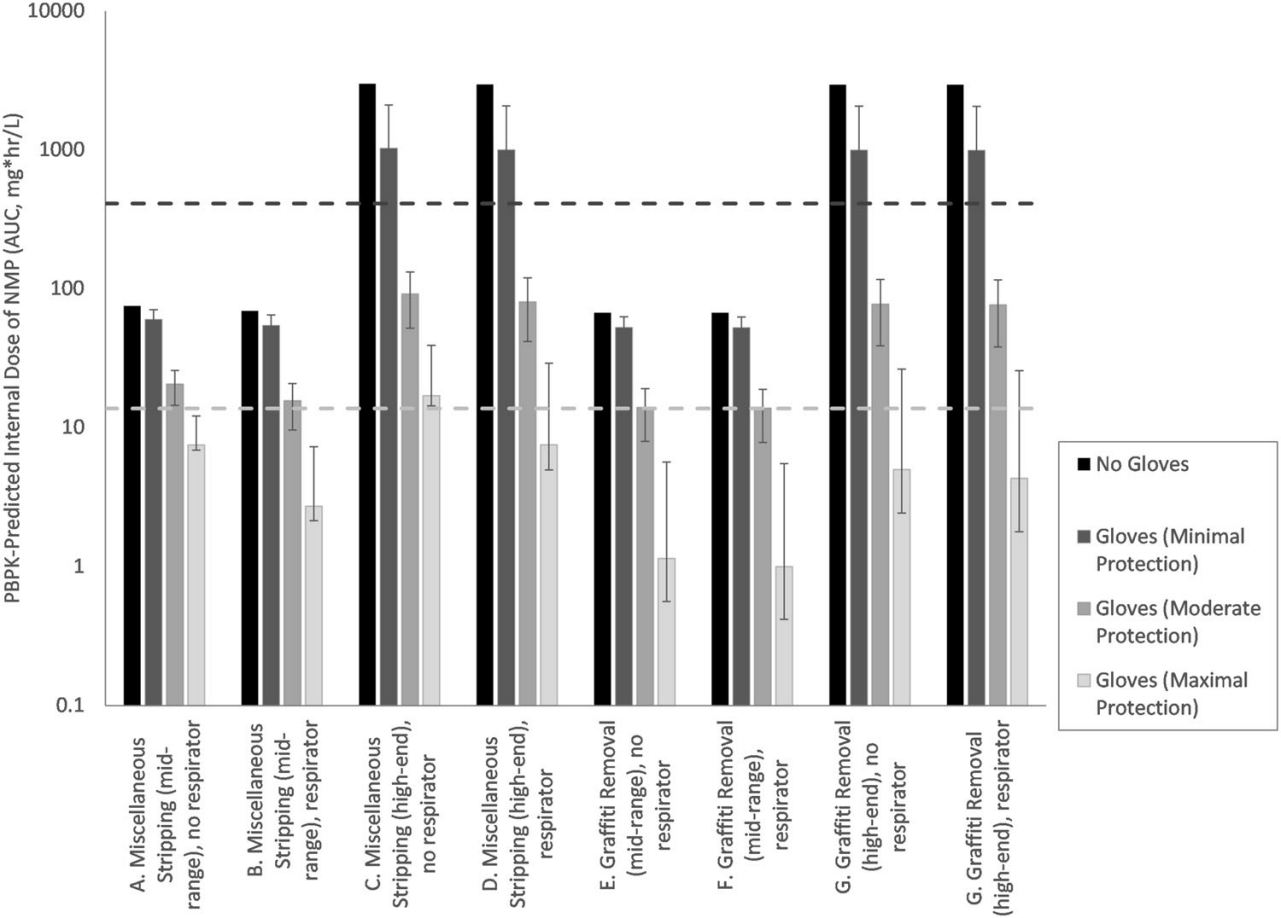
Internal dose estimates for chronic exposure scenarios using different glove types. Columns indicate the mean value for the glove category, error bars indicate the range for the glove category (based on the range of Kp_net_ values).

MOE results for the chronic exposure scenarios are provided in Fig. 2. MOE values calculated for moderate and maximum protection glove categories again exhibit some improvement over the no-glove values calculated by USEPA, while those calculated for minimum protection glove categories were minimally changed. Specifically, MOE values (rounded to 2 significant figures) calculated by USEPA for no glove scenarios ranged from 0.1 to 6.1, while the mean MOE values calculated for use of minimal, moderate, and maximum protection glove types range across scenarios from 0.40 to 7.9, 4.5 to 30, and 24 to 410, respectively.

For both acute and chronic scenarios, by greatly reducing the contribution of the dermal liquid pathway to total internal dose, the refined MOE values for the maximal protection glove groups are driven primarily by the inhalation and dermal vapor pathways (i.e., glove use does not affect internal dose predictions arising from these pathways).

Glove protection factors calculated from PBPK simulations (isolated for the dermal liquid pathway) performed for the eight exposure scenarios indicate that the degree of protection to NMP permeation offered by gloves varies by several orders of magnitude, and depends on glove material, NMP formulation (NMP solution vs. neat NMP), and measure of internal dose (Cmax vs AUC) (Table 4).

## Discussion/conclusion

A refined risk assessment was conducted to assess the efficacy of different glove materials in reducing the potential hazards associated with use of NMP-containing paint strippers. For acute exposure scenarios, gloves from the moderate protection group (polyethylene) offered sufficient protection for half of the scenarios assessed here, while gloves from the maximum protection group (laminate, butyl) offered sufficient protection for all scenarios. Gloves from the minimum protection group offer minimal protection when used on a task-specific basis (e.g., short-term splash protection for acute consumer scenarios). Furthermore, their use cannot be recommended due to their risk of acute failure (swelling, splitting of material) [4], a factor not specifically evaluated in this assessment. For chronic exposures to NMP-containing paint strippers, only gloves from the maximum protection group provided sufficient protection to workers for all scenarios except one, in which an MOE of 24 was calculated [Miscellaneous Stripping (high-end), no respirator]. The MOE value for this scenario is considered to approach a value of 30, and as discussed by Poet et al. [7] an MOE value of 21 may be considered adequately protective of a healthy worker population when a data-derived extrapolation factor for human toxicokinetic variation is adopted for NMP (see intraspecies variation discussion below).

The Kp_net_ values derived in this assessment reflect a relatively simple approach for incorporating the best available data for NMP glove permeation from in vitro studies. Future in vivo studies that characterize the absorbed dose of NMP in humans, both with and without gloves made from different materials, would be valuable in validating, refining, and/or replacing the approach taken in this assessment.

The MOE values calculated in this assessment are higher than calculated for the no-glove scenarios in USEPA’s TSCA risk assessment [1]. The adoption of a number of health protective assumptions embedded in the assessment provide confidence that the MOE values calculated remain conservative. These assumptions include:

- *Constant concentration of NMP in liquid on skin*—Consistent with the USEPA assessment, the concentration of NMP in liquid on skin or glove was assumed to be constant and infinite, rather than decrease over time due to absorption, volatilization, and transdermal flux of water [1]. This is a conservative assumption that is intended to be protective of repeated dermal exposure events; however for non-glove scenarios this assumption can result in large predicted volumes of NMP taken up by the skin (e.g., up to ~15 mL of NMP) over the course of a day. Modeling of the dermal liquid pathway as episodic in nature, with NMP concentrations decreasing over time or to amounts consistent with the use of finite volumes of strippers, is expected to result in lower, and more realistic exposure estimates.
- *Respirator efficacy*—The range of respirator efficacies at reducing inhalation exposures to NMP was not evaluated in this assessment. Instead, USEPA’s assumption of a 90% reduction in the air concentration was maintained for this assessment. Like glove permeation rate, the efficacy of respirators is expected to vary. Bader et al. [14] assessed the efficiency of the facemasks with activated carbon filtering to prevent the inhalation of NMP vapors. The authors reported that gas samples taken from behind the face shield masks show no NMP detected over an 8-h period of exposure to 80 mg/m^3^ (20 ppm), which suggests that the MOE values calculated here for respirator use scenarios may be underestimated for high efficacy respirators. However, a comparison of MOE values for scenarios with and without respirator show very similar results for scenarios without gloves (Figs. 1, 2), suggesting that inhalation of vapors was not a large contributor to total exposure in these scenarios. The relative importance of the inhalation pathway (and therefore the impact of respirator use) increases for scenarios with gloves, particularly for the maximal protection group where the contribution of the dermal liquid pathway is greatly reduced.
- *Prolonged dermal contact with NMP*—USEPA’s exposure scenarios for NMP included prolonged (up to 8 h) and repeated dermal contact with NMP. Because NMP is considered to be irritating to eyes and skin [15], prolonged and repeated dermal contact with NMP, as assumed in this assessment, may be self-limiting (e.g., behavior changes with respect to PPE use, increased attention in avoiding skin contact with liquid, and/or washing soon after contact).
- *Endpoint selection*—Because the endpoint selected for NMP risk assessment (developmental effects) are applicable to exposures to pregnant women, MOE values for male and non-pregnant female workers exposed to NMP are expected to be higher than those calculated here, since they would be based upon on a less sensitive endpoint (i.e., higher POD values for effects other than developmental effects).
- *Human PBPK model parameterization*—In developing their PBPK model for NMP, USEPA relied upon conservative parameter values for humans using only the low-concentration data from the human volunteer study of Bader et al. [16] (rather than rely upon data from all concentration levels). This approach results in more conservative estimates for internal dose in humans by ~1.3- to 1.4-fold [7]. This change alone would result in chronic MOE values >30 for 3/8 scenario for gloves from the moderate protection category, and for all eight scenarios for gloves from the maximum protection category.
- *Rat PBPK model parameterization*—The rat PBPK model for inhalation exposures to NMP was parameterized based upon a study for nose-only exposures [17], while the inhalation toxicity studies for NMP involved whole-body exposures. For this reason, the internal dose estimates predicted by the PBPK model for inhalation POD values may be underestimated (i.e., thereby overestimating its toxic potency), since they do not include contribution for additional exposure pathways: (1) dermal uptake of NMP vapors, which has been shown to be significant for NMP in humans [15] and vapor permeability for other volatile chemicals is approximately two to fourfold higher in rat skin compared with human skin [18]; and (2) oral dosing from grooming of NMP vapor adsorbed to rat fur, which has been shown to be significant for other chemicals [18–20].
- *Intraspecies variation*—An acceptable MOE value of 30 was defined by USEPA [1] for NMP, based upon consideration of interspecies differences in toxicodynamics (factor of 3), and intraspecies differences in toxicokinetics and toxicodynamics (factor of 10). However, an evaluation of human variation in toxicokinetics for NMP based on data from Bader et al. [17] suggests that MOE values of 20–21 (i.e., replacing a default factor of 3 for toxicokinetic variation, with a data-derived value of 2–2.1) may be considered protective for 95% of individuals from a healthy worker population [7]. A PBPK model used in USEPA’s assessment for methylene chloride revealed a similar approximately twofold range between average and lower percentile for human variation associated with toxicokinetic factors [21], This change alone would result in acceptable chronic MOE values for 3/8 scenarios for moderately protective gloves and 8/8 scenarios for maximally protective gloves.

- *Benchmark response rate*—For the acute assessment, the use of a benchmark response rate of 1% for developmental effects is lower than has been selected for other chemicals, which typically rely upon a benchmark response rate of 5% or equivalent to one standard deviation. In this case, use of benchmark response rate of one standard deviation would results in an acute POD (ID_TA_) and corresponding MOE values that are ~2.5-fold higher than those calculated here. Similarly, use of a benchmark response rate of one standard deviation would result in a 1.1-fold change in chronic POD and MOE values. This change alone would result in acceptable acute MOE values for all scenarios for glove from the moderately and maximally protective categories, while chronic MOE value conclusions would remain unchanged.
- *Exposure duration concordance*—There is some degree of discordance in the exposure durations used in acute toxicity and acute exposure assessments conducted for NMP. Specifically, the point of departure for acute endpoints relies upon observations following a 15-day exposure to NMP, which covers the majority of the rat gestation period (21 days). On the other hand, the exposure duration assumed for acute exposures to workers (1 day) reflects a small fraction of the human gestation period (40 weeks). Based upon a consideration of this issue for chemicals in general [22] and on NMP-specific data for the importance of exposure duration in producing fetal resorptions in mice exposed to NMP for durations of 1, 5, or 14 days of gestation [23], a one-day exposure to NMP is expected to be significantly higher (e.g., ~twofold) to produce an equivalent response for a 15-day exposure. This change alone would result in acceptable acute MOE values for all scenarios for glove from the moderately and maximally protective categories.

Refinements to the NMP risk assessment that address combinations of these conservative assumptions using probabilistic methods would be expected to result in MOE values that are considerably higher than calculated in this assessment, by perhaps as much as an order of magnitude. Such refinements would be consistent with USEPA’s definition for Reasonable Maximum Exposure (RME) [24], which should contain an appropriate mixture of upper-bound and average values for exposure assumptions.

The results of this refined risk assessment indicate that NMP-containing paint strippers can be used safely, provided that appropriate PPE (i.e., gloves made of NMP-resistant materials in the maximum protection category) are used. In this assessment, use of gloves from the maximum protection category reduced internal dose estimates of NMP following acute and chronic exposures by more than 90%, indicating that the dermal absorption of liquid NMP is the most important pathway contributing to total exposure to workers in the eight scenarios evaluated. These results can be used to support risk-reduction methods as pragmatic alternatives to banning the use of NMP paint strippers under TSCA, including better instructions (for inclusion in MSDS, product labeling) regarding the use of appropriate glove material when using NMP paint strippers.
